# piRNA-associated proteins and retrotransposons are differentially expressed in murine testis and ovary of aryl hydrocarbon receptor deficient mice

**DOI:** 10.1098/rsob.160186

**Published:** 2016-12-21

**Authors:** Eva M. Rico-Leo, Nuria Moreno-Marín, Francisco J. González-Rico, Eva Barrasa, Cristina Ortega-Ferrusola, Patricia Martín-Muñoz, Luis O. Sánchez-Guardado, Elena Llano, Alberto Alvarez-Barrientos, Ascensión Infante-Campos, Inmaculada Catalina-Fernández, Matías Hidalgo-Sánchez, Dirk G. de Rooij, Alberto M. Pendás, Fernando J. Peña, Jaime M. Merino, Pedro M. Fernández-Salguero

**Affiliations:** 1 Departamento de Bioquímica y Biología Molecular, Facultad de Ciencias, Universidad de Extremadura, Badajoz, Spain; 2 Departamento de Anatomía, Biología Celular y Zoología, Facultad de Ciencias, Universidad de Extremadura, Badajoz, Spain; 3 Servicio de Técnicas Aplicadas a las Biociencias (STAB), Universidad de Extremadura, Badajoz, Spain; 4 Laboratorio de Reproducción y Espermatología Equina, Hospital Veterinario, Universidad de Extremadura, Cáceres, Spain; 5 Departamento de Fisiología, Universidad de Salamanca, Salamanca, Spain; 6 Instituto de Biología Molecular y Celular del Cáncer (CSIC-Universidad de Salamanca), Salamanca, Spain; 7 Servicio de Anatomía Patológica, Hospital Universitario Infanta Cristina, Badajoz 06074, Spain; 8 Reproductive Biology Group, Division of Developmental Biology, Department of Biology, Faculty of Science, Utrecht University, Utrecht, The Netherlands

**Keywords:** aryl hydrocarbon receptor, nuage proteins, spermatogenesis, ovary, fertility, repetitive elements

## Abstract

Previous studies suggested that the aryl hydrocarbon receptor (AhR) contributes to mice reproduction and fertility. However, the mechanisms involved remain mostly unknown. Retrotransposon silencing by Piwi-interacting RNAs (piRNAs) is essential for germ cell maturation and, remarkably, AhR has been identified as a regulator of murine *B1-SINE* retrotransposons. Here, using littermate *AhR^+/+^* and *AhR*^−/−^ mice, we report that AhR regulates the general course of spermatogenesis and oogenesis by a mechanism likely to be associated with piRNA-associated proteins, piRNAs and retrotransposons. piRNA-associated proteins MVH and Miwi are upregulated in leptotene to pachytene spermatocytes with a more precocious timing in *AhR*^−/−^ than in *AhR^+/+^* testes. piRNAs and transcripts from *B1-SINE*, *LINE-1* and *IAP* retrotransposons increased at these meiotic stages in AhR-null testes. Moreover, *B1-SINE* transcripts colocalize with MVH and Miwi in leptonema and pachynema spermatocytes. Unexpectedly, *AhR*^−/−^ males have increased sperm counts, higher sperm functionality and enhanced fertility than *AhR^+/+^* mice. In contrast, piRNA-associated proteins and *B1-SINE* and *IAP*-derived transcripts are reduced in adult *AhR*^−/−^ ovaries. Accordingly, AhR-null female mice have lower numbers of follicles when compared with *AhR^+/+^* mice. Thus, AhR deficiency differentially affects testis and ovary development possibly by a process involving piRNA-associated proteins, piRNAs and transposable elements.

## 1. Background

The aryl hydrocarbon/dioxin receptor (AhR) is now generally accepted to be an important regulator of cell and organ physiology, in particular with respect to the homeostasis of the liver, skin, cardiovascular and immune systems [1]. The reproductive system is an additional AhR target organ and changes in AhR expression and/or activation may affect its function [2,3]. Male and female reproductive systems are very sensitive to the effects of environmental toxins. Thus, AhR activation by the carcinogen 2,3,7,8-tetrachlorodibenzo-*p*-dioxin (TCDD) seems to affect the male reproductive system in the rat with a moderate reduction in sperm counts and epididymis weight [4–6]. Gestational administration of TCDD decreases the absolute epididymal and testis weight in wild-type (*AhR^+/+^*), but not in AhR-null (*AhR*^−/−^) animals [7]. In female mice, the AhR ligand dimethylbenz[*a*]anthracene (DMBA) enhances apoptotic germ cell death in primordial follicles of wild-type but not of AhR-deficient mice [8,9].

The effects of AhR in male and female reproductive systems are pleiotropic. AhR is expressed in different cell types of the mouse testis, and the generation of AhR-null mice has provided relevant information about the development and maturation of this organ [3]. AhR deficiency affects testis function by inducing an age-dependent reduction in testis weight and regression of the seminal vesicles and coagulating glands that is not evident until 24 weeks of age and that requires ageing to 52 weeks to affect half of the mice [10]. In addition, AhR deficiency has a variable impact on epididymal sperm production ranging from no effect [7] to a moderate reduction that appeared in 52-week-old mice [10]. The roles of AhR in the maintenance of Sertoli and germ cells of the seminiferous epithelium and in the function of Leydig cells and the epididymis have not yet been addressed in AhR-null mice, although indirect studies suggest that this receptor may contribute to sperm transit and steroidogenic function [10,11]. AhR is also relevant for the maturation and functioning of the ovary [3,12]. Histological evaluation of adult AhR-null female ovaries revealed lower numbers of pre-antral and antral follicles [13] that grew slower and had smaller diameter than those from wild-type mice when placed in culture [14]. Interestingly, such differences were not due to increased atresia or enhanced apoptosis in *AhR*^−/−^ follicles [15], although it could involve a reduction in steroidogenic hormone levels (e.g. oestradiol) [14] or in insulin signalling [16]. Altogether, these phenotypic differences impair the ovulatory potential and fertility in *AhR*^−/−^ female mice [3,12,17,18]. Despite these previous observations, there have been no subsequent studies investigating the molecular mechanisms through which AhR contributes to reproductive function under physiological conditions and how they may be deregulated in mice lacking receptor expression. Moreover, the data available do not explain how AhR modulates testis and ovary maturation in young fertile male and female mice.

The maturation of germline cells is very sensitive to the deleterious effects of active transposable elements whose silencing is particularly important during spermatogenesis and oogenesis [19]. The molecular complexes formed by Piwi-interacting RNAs (piRNAs) and piRNA-associated proteins protect the genomic stability of germ cells by counteracting the effects of transposon-derived transcripts [19–21]. The three mouse homologues of *Drosophila* Piwi proteins, namely Miwi, Mili and Miwi2, are germline-specific proteins essential for spermatogenesis [22–24]. These proteins are components of the nuage, a unique cellular structure that also contains the germ cell-specific DEAD-box RNA helicase mouse vasa homologue (MVH) [25]. The analysis of conditional null mice has revealed that Mili is expressed from primordial germ cells (12.5 days post-coitum (dpc)) to round spermatids (20 days postpartum (dpp)), Miwi 2 is restricted from prenatal 15.5 dpc to postnatal 3 dpp, whereas Miwi has a late expression pattern from pachytene (14 dpp) to the round spermatid stage (20 dpp) [26,27]. Therefore, nuage proteins appear essential for male germline development from the prenatal spermatogonia stage up to postnatal spermiogenesis [28]. In the mouse ovary, although MVH and Mili are not essential for oogenesis or fertility, they seem to be required for the control of transposon expression [29]. These studies suggest that nuage proteins contribute to the production of piRNAs that will ultimately silence transcriptionally or post-transcriptionally transposon-derived transcripts during the maturation of germ cells [19].

We have previously shown that AhR regulates the expression of certain forms of short interspersed nuclear elements (SINE) of the murine B1 retrotransposons subfamily [30]. Moreover, AhR-dependent transcription of a B1-SINE retrotransposon represses the expression of target genes that contained such an element in their upstream promoters [31], thus revealing a functional interaction between AhR, retrotransposon activation and the control of gene expression.

Based on these observations, we hypothesized that the reproductive phenotypes produced by AhR deficiency could be linked to an altered expression of transposable elements and to changes in the profile of piRNAs and piRNA-associated proteins. In this study, we show that AhR deficiency has organ- and developmental stage-dependent effects. Neonatal and young AhR-null testes have increased retrotransposon expression, enhanced piRNA production and higher levels of piRNA-associated proteins. The temporal pattern of these markers appears consistent with an accelerated leptonema to pachynema transition during the meiotic prophase of *AhR*^−/−^ germ cells. Neonatal and young *AhR*^−/−^ females have fewer numbers of follicles in the ovary and the ampulae, reduced transposon levels and diminished expression of nuage proteins MVH and Miwi, when compared with *AhR^+/+^* mice. We suggest that AhR may be involved in the piRNA-transposon pathway that modulates the timing of spermatogenesis and oogenesis in the testis and ovary. Although such a mechanism is likely to be integrated within a more complex phenotype caused by AhR depletion in testis and ovary, we provide evidence for a developmental process that involves the AhR-dependent control of small non-coding RNAs presumably regulating cell differentiation *in vivo*.

## 2. Material and methods

### 2.1. Mice and treatments

Wild-type (*AhR^+/+^*) and AhR-null (*AhR*^−/−^) littermate mice were in the C57BL/6N genetic background and were obtained from heterozygous crossings. Male and female mice were used between 2 and 24 days dpp or at the adult age of 10–12 weeks. For intratesticular injection, adult male mice were anaesthetized with an O_2_ + 3% isoflurane mix. The left testis was injected with 4 µg kg^−1^ of 6-formylindolo[3,2-b]carbazole (FICZ) diluted in a PBS solution containing 0.04% trypan blue to monitor accurate delivery of the AhR inducer. The right testis was injected with a PBS/0.04% trypan blue solution. After 8 h, mice were killed, and testes recovered for RNA analyses. ICR females at six to eight weeks of age were purchased from Harlan.

### 2.2. Antibodies and reagents

The following antibodies were used: anti-AhR clone RPT1 (Thermo Scientific, cat. no. MA1-514) for immunofluorescence and clone SA-210 (Biomol cat. no. BML-SA210) for western blotting, anti-MVH (AbCam cat. no. ab13840), anti-Mili (Thermo Scientific cat. no. PA5-17036) and anti-Miwi (Thermo Scientific cat. no. PA5-17034). The antibody for β-actin was from Sigma-Aldrich (cat. no. A-2066). The iScript™ Reverse Transcription Supermix was from Bio-Rad, and the SYBR^®^ Select Master Mix for real-time PCR (RT-qPCR) was from Life Technologies. The AhR agonist 6-formylindolo[3,2-b]carbazole (FICZ) was from Enzo. LIVE/DEAD Fixable Aqua Dead Cell Stain kit (Ex.: 405 nm, Em.: 525 nm) and Mitotracker Deep Red (Ex.: 644 nm, Em.: 665 nm) were purchased from Molecular Probes. The mouse steroidogenic factor-1 (SF-1) ELISA kit was from MyBiosource.

### 2.3. SDS–PAGE and immunoblotting

SDS–PAGE and immunoblotting were performed using total protein extracts obtained from testis and ovaries of *AhR^+/+^* and *AhR*^−/−^ mice as previously described [32]. Briefly, tissues were extracted, minced and homogenized in ice-cold lysis buffer (50 mM Tris–HCl pH 7.5, 150 mM NaCl, 0.5% Nonidet P-40, 1 mM phenyl-methyl sulfonyl fluoride, 1 mM NaF, 1 mM sodium orthovanadate, 1 mM DTT, 10 mM β-glycerophosphate and 4 µg µl^−1^ complete protease inhibitor cocktail). Following centrifugation at 15 000*g* for 30 min at 4°C, protein concentration was determined in the supernatants using the Coomassie Plus protein assay reagent (Pierce) and bovine serum albumin as standard. Aliquots of 20–30 µg of total protein were electrophoresed in 8% SDS–PAGE gels. Gels were transferred to nitrocellulose membranes that were subsequently blocked in a TBS-T solution (50 mM Tris–HCl pH 7.5, 150 mM NaCl, 0.2% Tween-20) containing 5% non-fat milk. Blots were incubated with the primary and secondary antibodies, washed in TBS-T and revealed using the Super-signal luminol substrate (Pierce).

### 2.4. RNA isolation and real-time RT-qPCR

Total RNA was isolated from mouse testis and ovary using the Trizol reagent (Life Technologies) followed by a DNAse treatment (Applied Biosystems). To analyse mRNA expression by RT-qPCR, total RNAs were further purified using the High Pure RNA isolation kit following the manufacturer's instructions (Roche). To analyse transposon transcripts and piRNAs by northern blotting and RNA labelling, isolated RNAs were extracted with phenol : chloroform (1 : 1), precipitated with ethanol and resuspended in DEPC-treated water. Reverse transcription was performed using random priming and the iScript Reverse Transcription Super Mix (Bio-Rad). Real-time PCR was used to quantify the mRNA expression of *AhR*, *MVH*, *Mili*, *Miwi, Cyp1a1* and *Gapdh,* as well as the levels of RNA transcripts from *SINE, B1-SINE, IAP* and *LINE-1* (*ORF2* and *5*′*UTR*) transposable elements. Reactions were done using SYBR^®^ Select Master Mix (Life Technologies) in a step one thermal cycler (Applied Biosystems) essentially as described [33]. *Gadph* was used to normalize gene expression (ΔCt) and 2^−ΔΔCt^ was applied to calculate changes in RNA levels with respect to control conditions. Representative RT-qPCR experiments were also normalized by β-actin to confirm reproducibility in the quantification of RNA expression levels. Primer sequences used are indicated in the electronic supplementary material, table S1.

### 2.5. Northern blotting and radioactive RNA labelling

RNA analysis by northern blotting was performed in *AhR^+/+^* and *AhR*^−/−^ testes at 8, 10, 12 and 14 dpp. Total RNA was electrophoresed in 8 M urea–polyacrylamide gels and transferred to Hybond-N^+^ nylon membranes (GE Healthcare). Hybridizations were performed overnight at 42°C in ULTRAhybR-oligo hybridization buffer (Ambion), using ^32^P-radiolabelled DNA oligonucleotides complementary to B1-SINE retrotransposons (see §3.2). After extensive washing in 2 × SSC/0.1% SDS at 60°C, membranes were exposed to X-ray film (Kodak) and developed. Radioactive RNA labelling for the detection of total piRNAs was performed, using 12, 14 and 24 dpp testes. RNA molecules were dephosphorylated at their 5′ ends with calf intestinal alkaline phosphatase (New England BioLabs) and end-labelled using T4 polynucleotide kinase (New England BioLabs) and [γ-^32^P]-ATP (6000 Ci mmol^−1^, Perkin Elmer). Aliquots of 0.5 to 1 µg of radiolabelled RNA were separated in 8 M urea–polyacrylamide gels, which were dried and exposed to a PhosphorImager screen (Bio-Rad). Sephadex G25 microspin columns (GE Healthcare) were used to purify oligonucleotide probes and labelled RNA. The size markers used correspond to a mix of DNA molecules of 20, 22, 53 and 100 nucleotides in length. The intensity of the radioactive bands was quantified by densitometry analysis, using ImageJ software. Band intensity in *AhR^+/+^* testis was considered equal to 1.0. For RNA analysis by ethidium-bromide staining, 50 µg of total RNA from 24 dpp *AhR^+/+^* and *AhR*^−/−^ testes was electrophoresed in agarose gels. After staining with ethidium bromide, gels were visualized by UV transillumination.

### 2.6. Immunohistochemistry

Testes and ovaries were collected, fixed in buffered formalin and embedded in paraffin. Tissues were sectioned at 3.5 µm, deparaffinated and rehydrated to PBS. Harris haematoxylin was added for 3 min at room temperature. After washing with tap water, eosin solution was added for 1 min. A final washing step was performed, and the tissues were dehydrated, mounted and observed under the microscope. For the quantification of spermatocyte-positive tubules, testes were collected, fixed in Bouin's solution and embedded in paraffin. Sections were prepared, stained with haematoxylin–PAS, mounted and observed under the microscope. Light microscopy was done at room temperature on a NIKON E-400 microscope equipped with a NIKON L16 camera. Objectives used were: 4× (0.10 numeric aperture), 10× (0.25 numeric aperture) and 20× (0.40 numeric aperture) on Mowiol mounted sections.

### 2.7. Immunofluorescence

Testis and ovary sections (3.5 µm) were deparaffinated and gradually re-hydrated to PBS. Non-specific epitopes were blocked by 1 h incubation in PBS containing 0.05% Triton X-100, 0.2% gelatin and 3% BSA. Sections were then incubated overnight at 4°C with the corresponding primary antibodies diluted in PBS-T/0.2% gelatin. Following washing in the same gelatin solution, sections were incubated for 1 h at room temperature with an Alexa-633 labelled secondary antibody. After additional washing, sections were dehydrated, mounted on Mowiol and visualized using an Olympus FV1000 confocal microscope (Olympus). Objectives used were: 10× (0.40 numeric aperture) and 20× (0.70 numeric aperture). Fluorescence analysis was done using the FV10 software (Olympus). DAPI was used to stain cell nuclei.

### 2.8. *In situ* hybridization

The expression of *B1-SINE* retrotransposons was analysed in cryostat sections of testes of *AhR^+/+^* and *AhR*^−/−^ mice at 12 and 14 dpp by *in situ* hybridization essentially as described [34]. In brief, testes were collected, frozen, postfixed in 4% paraformaldehyde and sectioned. After acetylation, permeabilization and washing, tissue sections were pre-hybridized for 2 h at room temperature in a buffer containing 50% formamide, 5 mM EDTA, 50 µg ml^−1^ yeast tRNA, 0.2% Tween-20 and 0.2% CHAPS. Digoxigenin-labelled RNA probes were diluted in pre-hybridization solution at a concentration of 200–300 ng ml^−1^, heated at 80°C for 5 min, cooled on ice and added to the tissue sections. Hybridization was performed overnight at 72°C in a humidified chamber. Sections were then sequentially washed in 0.2 x SSC at 72°C for 1 h and in 100 mM Tris–HCl (pH 7.5), 100 mM NaCl, 0.1% Triton X-100. Tissue sections were blocked in 0.1 M lysine monohydrochloride containing 10% normal goat serum and incubated overnight with alkakine phosphatase-conjugated anti-digoxigenin antibody. After washing, sections were incubated with nitroblue tetrazolium (NBT) and 5-bromo-4-chloro-3-indolyl phosphate (BCIP) as substrates (Roche). Samples were then washed in PBS and mounted using Mowiol. To prepare the sense and antisense probes detecting *B1-SINE* transcripts, the *B1-SINE* sequence was first amplified by PCR using the oligonucleotides indicated in the electronic supplementary material, table S1. The PCR product was then cloned in the pGEM-T easy vector for probe synthesis. The antisense probe was synthesized using T7 RNA polymerase in a *Pst*I-digested pGEM-T vector, whereas the sense probe was produced using SP6 RNA polymerase in an *Apa*I-digested pGEM-T vector. In some experiments, immunofluorescence for MVH, Mili or Miwi was performed in the same tissue sections following *in situ* hybridization. In those cases, primary antibodies were added before mounting and immunological detection completed as indicated above.

### 2.9. Epididymal sperm and fertility assays

Groups of 10 *AhR^+/+^* and *AhR*^−/−^ male mice of eight to 10 weeks of age were killed and their epididymides extracted and squeezed to recover the sperm into 1 ml of Pure Sperm Wash medium (Nidacon). The suspension was gently homogenized, and the number of sperm cells counted using a Neubauer chamber. Two independent observers taking at least eight independent measurements of every field counted each sample. Only motile cells were considered. The fertility of *AhR^+/+^* and *AhR*^−/−^ male mice was assessed by breeding experiments with fertile ICR females of six to eight weeks of age (Harlan). Groups of 20 ICR females were mated with males (at least four mice of each genotype) and the number of viable pups recovered at 18 dpc.

### 2.10. Functional characterization of epididymal spermatozoa

For a functional characterization, epididymides from eight *AhR^+/+^* and *AhR*^−/−^ mice were isolated and used to obtain sperm preparations in TNE medium essentially as described [35]. Spermatozoon suspensions were then divided into two fractions. One was processed for the analysis by flow cytometry of mitochondrial activity and membrane integrity.

### 2.11. Steroidogenic activity

SF-1 levels were quantified in *AhR^+/+^* and *AhR*^−/−^ male mice serum using a commercially available ELISA kit (MyBiosource) following the manufacturer's instructions. Briefly, blood was collected from mice in EDTA-treated tubes, gently homogenized and rapidly centrifuged at 5000*g* for 5 min at 4°C. After a second centrifugation under the same conditions, the supernatant was stored at −80°C. Aliquots of 50 µl of serum from each mouse were added in triplicate to each well of the kit and the procedure developed as indicated. Optical density was measured at 450 nm in a Varioskan Flash instrument (Thermo Scientific) and the concentration of serum SF-1 determined by using a standard curve prepared with known amounts of purified SF-1.

### 2.12. Flow cytometry

The following stock solutions were prepared: LIVE/DEAD Fixable Aqua Dead Cell Stain kit (50 µl of DMSO in the LIVE/DEAD vial) and MitoTracker Deep Red (500 µM in DMSO). Samples of 1 ml containing 1 × 10^6^ spermatozoa ml^−1^ in PBS were stained with 1 µl of LIVE/DEAD Fixable Aqua Dead Cell solution and 0.3 µl of MitoTracker Deep Red. After through mixing, the samples were incubated at room temperature (22°C) for 30 min in the dark. Then, spermatozoa were washed in PBS before analysis in the flow cytometer. Flow cytometry analysis was conducted, using a MACSQuant^®^ Analyser 10 (Miltenyi Biotech) equipped with three lasers emitting at 405, 488 and 635 nm and 10 photomultiplier tubes (PMTs). The cytometer was controlled using MACSQuantify^®^ software 2.6. Sperm subpopulations were either evaluated by histograms or divided by quadrants (density plots) to quantify the frequency of each subpopulation. A total of 40.000 events were analysed in each sample. The system was calibrated daily, using specific calibration beads provided by the manufacturer. Although spectral overlap with the probes used was unlikely, compensation of spectral overlap was performed before each experiment using negative (unstained) and positive (single-stained) controls for each single-stained compensation control sample. Limits for each quadrant were determined using unstained and single-stained control samples. The data were analysed using FlowJo v. 10 software (FlowJo, LLC).

### 2.13. Sperm motility and kinematic parameters

The second fraction of the sperm preparation was gently centrifuged, resuspended in HBSS medium supplemented with 2% BSA and incubated at 37°C for 30 min. These samples were used to measure circular (VCL), linear (VSL) and average (VAP) spermatic velocities as well as the percentages of total motile (TM) and progressive motile (PM) spermatozoa. Sperm motility and kinematic parameters were assessed using a computer-assisted sperm snalysis (CASA) system (ISAS Proiser). Semen (3 µl) was loaded in 20 µm depth Leja^®^ chambers (Leja) and placed on a 37°C warmed stage. The analysis was based on the evaluation of 60 consecutive digitalized images per second, using a 10× negative phase-contrast objective (Olympus CX41). At least three different fields were recorded to ensure that no less than 200 spermatozoa were analysed per sample. Spermatozoa with a VAP < 10 µm s^−1^ were considered immobile, whereas spermatozoa with VAP > 15 µm s^−1^ were considered motile. Spermatozoa deviating less than 75% from a straight line were catalogued as linearly motile and spermatozoa with a VCL > 45 µm s^−1^ were designated as rapid sperm.

### 2.14. Isolation of follicles, germinal vesicles and MII oocytes

*AhR^+/+^* and *AhR*^−/−^ female mice of eight to 12 weeks of age were killed and their ovaries extracted. Fourteen mice of each genotype were used to isolate follicles and germinal vesicles (GV), of which 10 mice per genotype were used to obtain oocytes at the MII developmental stage. Ovaries were gently disaggregated using forceps in MEM medium supplemented with 25 mM HEPES, 0.23 mM sodium pyruvate, 3 mg ml^−1^ BSA and 1% penicillin–streptomycin. Follicles were separated from GVs using a stripper (Origio), and each sample group was independently fixed in 3.5% paraformaldehyde for 15 min at room temperature. The zona pellucida was removed by incubation in Tyrode's acid solution for 15–20 s at 37°C. For immunofluorescence analysis, oocytes were blocked in PBS containing 1% BSA and 0.1 M glycine for 1 h. Follicles and GVs were incubated in blocking solution with antibodies against MVH, Mili or Miwi overnight at 4°C. Following washings, an Alexa-633-labelled secondary antibody was added for 2 h at 4°C. Samples were further washed and incubated with DAPI to stain cell nuclei. Oocytes were transferred to Ibidi chambers and analysed using an Olympus FV1000 confocal microscope. MII stage oocytes were isolated from the ampulae of the oviduct and mechanically dissociated from the follicular cells with a stripper (Origio). They were subsequently fixed and processed for immunofluorescence as indicated above.

### 2.15. Statistical analyses

Data are shown as means ± s.d. Comparisons between experimental conditions were done using GraphPad Prism v. 6.0 software (GraphPad). The student's *t*-test was used to analyse differences between two experimental groups and ANOVA for the analyses of three or more groups. The Mann–Whitney non-parametric statistical method was used to compare rank variations between independent groups.

## 3. Results

### 3.1. Aryl hydrocarbon receptor deficiency increases the levels of nuage and piRNA-associated proteins in the testis

**Figure 1.**
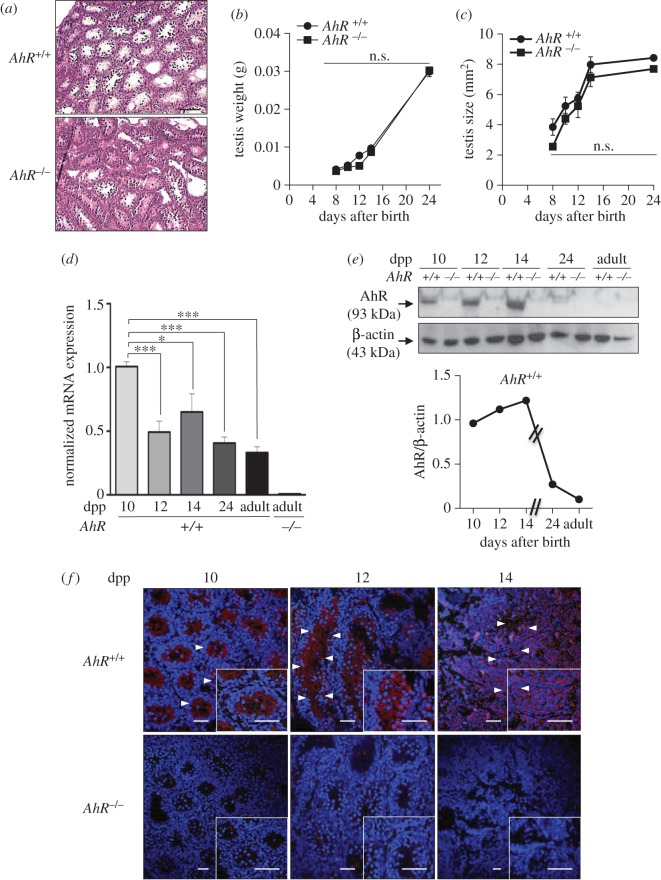
AhR is expressed in mouse testis and it does not significantly affect testis weight or size. (*a*) Testes from 24 dpp *AhR^+/+^* and *AhR*^−/−^ mice were extracted, processed histologically and stained with haematoxylin–eosin. (*b*,*c*) Testes weight (*b*) and size (*c*) were determined in mice of each genotype at the indicated times after birth. Testis size was calculated using the formula [length × width^2^ × 0.4]. (*d*) *AhR* mRNA expression was quantified by RT-qPCR in *AhR^+/+^* testes at the indicated ages, using total RNA and the specific primers indicated in Material and methods. RNA isolated from adult *AhR*^−/−^ testes was used as negative control. RT-qPCR data were normalized by the expression of *Gapdh* and represented as 2^−ΔΔCt^. (*e*) AhR protein expression was determined by immunoblotting in *AhR^+/+^* testes at the indicated ages using total protein extracts. The expression of β-actin was used to normalize protein loading. The graph represents the profile of AhR protein expression over time. (*f*) Immunofluorescence analysis of AhR expression in *AhR^+/+^* and *AhR*^−/−^ mouse testes at 10, 12 and 14 dpp. Arrowheads mark the presence of AhR (red fluorescence) in the seminiferous tubules. Testes extracted from *AhR*^−/−^ mice were used as negative controls. DAPI was used to stain cell nuclei. Details of the micrographs are shown in the insets. Four biological replicates and two experimental duplicates were done for panels (*d,e*). At least four individual mice of each genotype were used for the rest of the experiments. Data in panels (*b–d*) are shown as mean ± s.d. *p < 0.05. n.s. not statistically significant. Adult mice were 12–13 weeks of age. Scale bar, 50 µm.

Previous studies have shown that AhR-null mice have an altered seminiferous epithelium [36] and seminal vesicle regression that only become apparent with ageing over 52 weeks and that was undetectable in younger mice [10]. However, the molecular pathways through which AhR participates in the homeostasis of the male reproductive system are mostly unknown. Young *AhR*^−/−^ mice [37] of the C57BL/6N genetic background had neither significant histological alterations in their seminiferous tubules nor a reduction in testis weight/size with respect to littermate *AhR^+/+^* mice (figure 1*a*–*c*). In testes from neonate to adult wild-type mice, AhR mRNA levels gradually decreased with an apparent increase in expression at 14 dpp (figure 1*d*). The AhR protein profile also showed a moderate increase until 14 dpp to sharply decrease at 24 days dpp and later on in adults (figure 1*e*). Immunological analysis revealed that AhR expression in the seminiferous tubules of *AhR^+/+^* male mice that was largely restricted to internal layers of cells at 10–12 dpp changed to a more generalized localization at 14 dpp (figure 1*f*). On the contrary, testis from *AhR*^−/−^ mice did not have any detectable levels of AhR mRNA (figure 1*d*) or protein (figure 1*e,f*).

**Figure 2.**
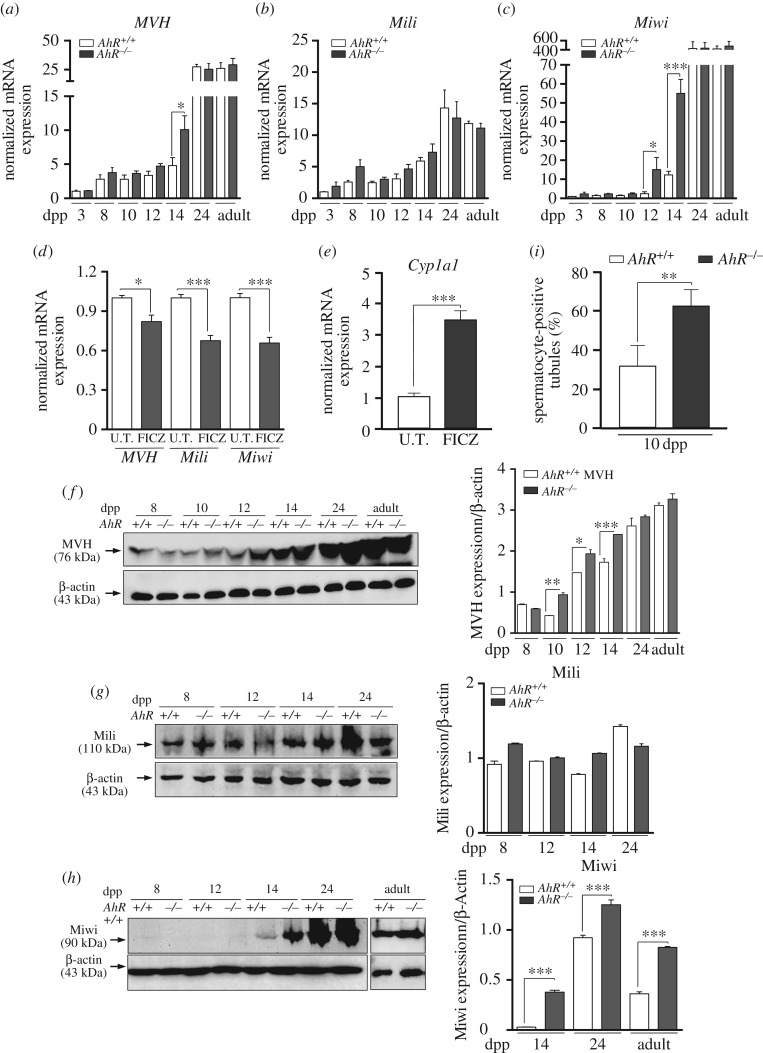
AhR modulates the expression of piRNA-associated proteins MVH, Mili and Miwi. (*a*–*c*) Testes were obtained from *AhR^+/+^* and *AhR*^−/−^ mice at 3, 8, 10, 12, 14 and 24 dpp and from adults and used to isolate RNA. mRNA expression for *MVH* (*a*), *Mili* (*b*) and *Miwi* (*c*) was determined by RT-qPCR, using the oligonucleotides indicated in Material and methods. (*d*) *AhR^+/+^* mice were subjected to intratesticular injection of 4 µg kg^−1^ of 6-formylindolo[3,2-b]carbazole (FICZ) to activate AhR *in vivo* and the mRNA expression of *Mvh*, *Mili* and *Miwi* analysed by RT-qPCR. (*e*) The efficiency of FICZ to induce AhR in the testis was analysed by measuring the mRNA expression of its canonical target gene *Cyp1a1*. Gene expression in panels (*a–e*) has been normalized by *Gapdh* and represented as 2^−ΔΔCt^. (*f–h*) Testes from *AhR^+/+^* and *AhR*^−/−^ mice were obtained and total protein extracts prepared and analysed for the expression of MVH (*f*), Mili (*g*) and Miwi (*h*) by immunoblotting using specific antibodies. β-Actin was used to normalize protein levels. (*i*) The maturation of *AhR^+/+^* and *AhR*^−/−^ testes at 10 dpp was determined by the microscopic quantification of spermatocyte-positive seminiferous tubules in at least six different male mice of each genotype. At least four individual mice of each genotype were used for the experiments shown in panels (*a–h*). Determinations were done in duplicate. Data are shown as mean ± s.d. *p <0.05, **p < 0.01, ***p < 0.001. Adult mice were 12–13 weeks of age.

Nuage proteins are considered essential for a normal spermatogenesis to take place. *MVH* and *Mili* are widely expressed in mouse germ cells from embryonic day E10.5 to close to the round spermatid stage at 20 dpp [25–27]. *Miwi* has a more restricted expression profile and is present from pachytene spermatocytes (14 dpp) to round spermatids (20 dpp) [26]. Real-time PCR analysis showed that *MVH* mRNA significantly increased in 14 dpp *AhR*^−/−^ pachytene spermatocytes (figure 2*a*). *Mili* mRNA levels were essentially unaffected by AhR expression within the same timeframe (figure 2*b*), whereas *Miwi* had an expression pattern similar to that of *MVH* despite its negligible levels before 12 dpp (figure 2*c*). In agreement with the role of AhR in regulating the expression of these nuage-dependent proteins, *in vivo* intratesticular injection of the AhR non-toxic ligand 6-formylindolo[3,2-b]carbazole (FICZ) significantly repressed *MVH*, *Mili* and *Miwi* mRNA expression in *AhR^+/+^* mice (figure 2*d*). Controls were performed to confirm the ability of FICZ to activate AhR in testis *in vivo* by measuring the induction of its canonical target gene *Cyp1a1* [38] (figure 2*e*). Protein expression patterns for MVH, Mili and Miwi were close to those found at the mRNA level. MVH protein levels were higher in *AhR*^−/−^ than in *AhR^+/+^* testis from 10 to 14 dpp and in the adult age (figure 2*f*). Mili had similar protein expression levels in testes of both genotypes from 8 to 24 dpp (figure 2*g*). In agreement with the mRNA expression profile, Miwi protein remained undetectable until 12 dpp in both genotypes but experienced a significant increase from 14 dpp to the adult stage, more notably in *AhR*^−/−^ testes (figure 2*h*). These data suggest that *AhR*^−/−^ testes have an altered maturation process. To determine the time point at which the lack of AhR influences testis development, testes from *AhR^+/+^* and *AhR*^−/−^ mice were analysed for the presence of spermatocyte-positive seminiferous tubules. As shown in figure 2*i*, AhR-null mice had a significant increase in spermatocyte-containing tubules at 10 dpp with respect to wild-type mice, suggesting that testis maturation could take place earlier in *AhR*^−/−^ than in *AhR^+/+^* mice.

**Figure 3.**
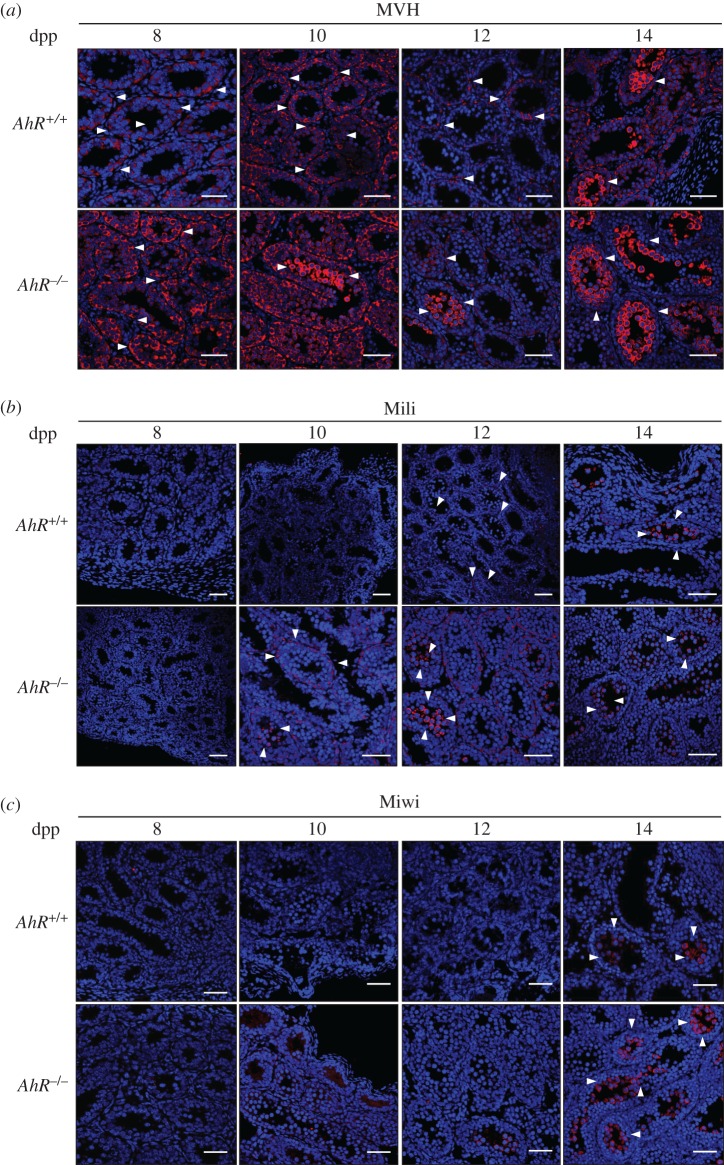
AhR deficiency may accelerate the temporal expression pattern of MVH, Mili and Miwi. *AhR^+/+^* and *AhR*^−/−^ testes were extracted at 8, 10, 12 and 14 dpp and processed for immunohistochemistry as indicated in Material and methods. (*a*) Testis sections were stained for MVH using a specific antibody. (*b*) Sections were also analysed for the location of the Mili protein. (*c*) The pattern of Miwi expression was also determined in testis sections of both genotypes. DAPI staining was used to label cell nuclei. Arrowheads indicate protein expression. At least five individual mice of each genotype were used for the experiments, and different sections from each testis were analysed. Scale bar, 50 µm.

We next used immunofluorescence to analyse if the lack of AhR modifies the distribution of MVH, Mili and Miwi in the seminiferous tubules. In wild-type testes, MVH was expressed almost exclusively in the basal compartment of the seminiferous tubules in spermatogonia until 12 dpp; at 14 dpp, spermatocytes clearly also expressed MVH (figure 3*a*, top). In AhR-null testes, MVH was highly expressed in the outer layer of basal spermatogonia at 8 dpp, but as early as 10 dpp spermatocytes also stained for MVH, and this was also seen at 14 dpp (figure 3*a*, bottom). Mili levels were markedly lower in both genotypes but, as observed for MVH, it was mainly present in the spermatogonia layer at 12 dpp and in the spermatocyte compartment at 14 dpp in *AhR^+/+^* testes (figure 3*b*, top). In *AhR*^−/−^ testes, Mili already localized to the spermatocyte compartment at 12 dpp (figure 3*b*, bottom). Miwi, which appears at pachynema in mice [26], was present in the spermatocyte compartment at 14 dpp in both genotypes, being more abundant in *AhR*^−/−^ than in *AhR^+/+^* seminiferous tubules (figure 3*c*). Thus, AhR deficiency may accelerate the temporal expression profile of MVH, Mili and Miwi along the differentiation process of the mouse testis.

### 3.2. Aryl hydrocarbon receptor modulates transposons and piRNAs levels during testis maturation

**Figure 4.**
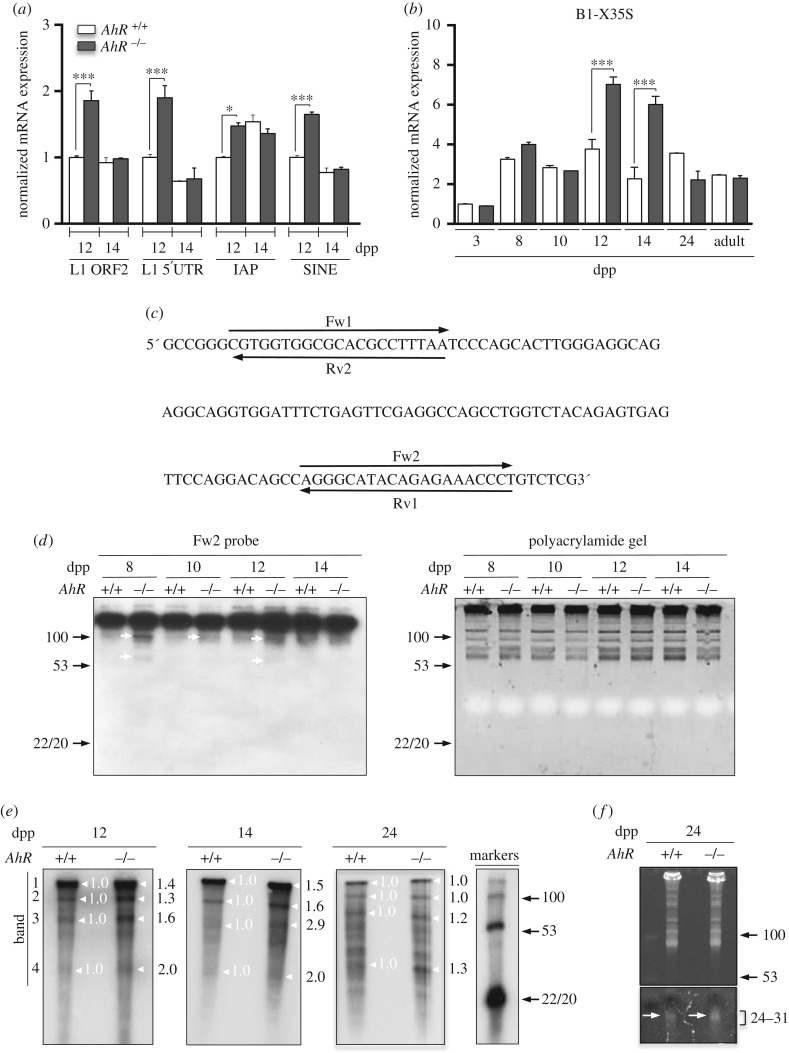
Testes lacking AhR have increased expression of transposons and higher levels of piRNAs. (*a*) *AhR^+/+^* and *AhR*^−/−^ testes obtained at 12 and 14 dpp were used to purify RNA as indicated in Material and methods. The expression of *L1-ORF2*, *L1-5′UTR*, *IAP* and *B1-SINE* transposons was quantified by RT-qPCR. (*b*) The same analysis was done for the *B1-X35S* retrotransposon using testes at 3, 8, 10, 12, 14 and 24 dpp and from adult mice. Gene expression in panels (*a*) and (*b*) has been normalized by *Gapdh* and represented as 2^−ΔΔCt^. (*c*) Sequence analysis was performed on *B1-SINE* retrotransposons in order to design hybridization probes for northern analysis. (*d*) Northern analysis was done using RNA purified from *AhR^+/+^* and *AhR*^−/−^ testes at 8, 10, 12 and 14 dpp. Arrowheads indicate the potential transposon-derived transcripts. Molecular sizes are indicated in nucleotides. Only the Fw2 probe hybridized with the RNA. Gels used for northern blotting were previously stained with ethidium bromide to verify equal loading and integrity of the RNA (right). (*e*) RNA labelling was used to determine differences in piRNA levels between *AhR^+/+^* and *AhR*^−/−^ testes at 12, 14 and 24 dpp. White arrows indicate the position of piRNAs and standard DNA sizes are shown in nucleotides in the markers lane on the right. Major bands (1–4) present in 12, 14 and 24 dpp have been quantified using ImageJ software. Expression levels in *AhR^+/+^* testis have been normalized to a value of 1.0. (*f*) Testes from 24 dpp mice of both genotypes were also analysed for the presence of piRNAs by agarose gel electrophoresis. Gels were stained with ethidium bromide and photographed. piRNAs are indicated by arrows. Molecular sizes are shown in nucleotides. The same gel is shown at two different exposures to show the presence of piRNAs (lower, 24–31 nucleotides) and equal loading (upper). Five biological replicates were done and at least two experimental replicates. Data in panels (*a,b*) are shown as mean ± s.d. *p < 0.05, ***p < 0.001. Adult mice were 12–13 weeks of age. Note that DNA migrates 10% faster than RNA in a 15% polyacrylamide gel.

Nuage and piRNA-associated proteins regulate the production of piRNAs that will drive silencing of transposons during maturation of germ cells [24,39,40]. Thus, we next investigated whether the pattern of nuage and piRNA-associated proteins observed in *AhR*^−/−^ testes correlated with an altered expression of transposons and piRNAs. Several transposon families are transcribed in the germline including *LINE1* (transcripts from their second open reading frame *ORF2* (*L1-ORF2*) and their *5'UTR* region (*L1–5′UTR*)), *IAP* (intracisternal A-type particle 1) and *B1-SINEs* [22,29,41]. The analysis of these elements showed that their expression was significantly increased in *AhR*^−/−^ testis during the zygonema to pachynema transition at 12 dpp, but remained essentially unaffected by the presence of AhR at 14 dpp (figure 4*a*). Within the *B1-SINE* family of transposable elements, we decided to analyse *B1-X35S,* because it is known to be regulated by AhR [30]. Indeed, AhR-null testes had a significant increase in *B1-X35S* transcription at 12 and 14 dpp with respect to *AhR^+/+^* testes (figure 4*b*). Sequence analysis allowed the design of different probes (Fw1, Fw2, Rv1 and Rv2) in order to identify *B1-SINE*-derived non-coding RNA transcripts at the times when increased expression of nuage proteins was detected in testis (figure 4*c*). Preliminary RNA analysis revealed that only the Fw2 probe hybridized with RNAs extracted from *AhR^+/+^* and *AhR*^−/−^ testes (not shown). Northern blot analysis using the Fw2 probe showed that while transcripts of more than 100 nucleotides in length remained essentially unchanged between genotypes and during spermatocyte maturation, small RNA molecules possibly representing processed *B1-SINE* transcripts with sizes ranging from 60 to 100 nucleotides were more abundant in *AhR*^−/−^ than in *AhR^+/+^* testes at 8, 10 and 12 dpp (figure 4*d*, left). Polyacrylamide gels used in these experiments were previously stained with ethidium bromide to confirm similar loading of RNA among the different experimental conditions analysed (figure 4*d*, right). We next used radioactive RNA labelling to examine the presence of piRNAs in *AhR^+/+^* and *AhR*^−/−^ testes. AhR deficiency increased the amounts of piRNA molecules with sizes close to 30 nucleotides at 12 and 14 dpp with respect to *AhR^+/+^* mice (figure 4*e*, white arrows). Longer RNA molecules were also observed in both genotypes that could possibly correspond to precursor RNA transcripts that were also identified in previous studies [39]. Quantification of signal intensity of the four major bands present in the radioactive gels revealed that *AhR*^−/−^ testis had increased levels of molecules within the piRNA size (figure 4*e*, band 4) and of longer RNAs of up to 100 nucleotides in length at 12, 14 and 24 dpp (figure 4*e*, bands 2 and 3). Moreover, an increase in short piRNAs in the range of 24–31 nucleotides was detected in 24 dpp *AhR*^−/−^ testis corresponding to the round spermatid stage of spermatozoa differentiation (figure 4*f*). Combining these results with the radioactive analysis of non-coding RNAs, it appears that both longer precursor molecules (figure 4*e*, white arrows) as well as short processed piRNA transcripts of 24–31 nucleotides were produced at higher amounts in *AhR*^−/−^ testis (figure 4*f*, arrows).

**Figure 5.**
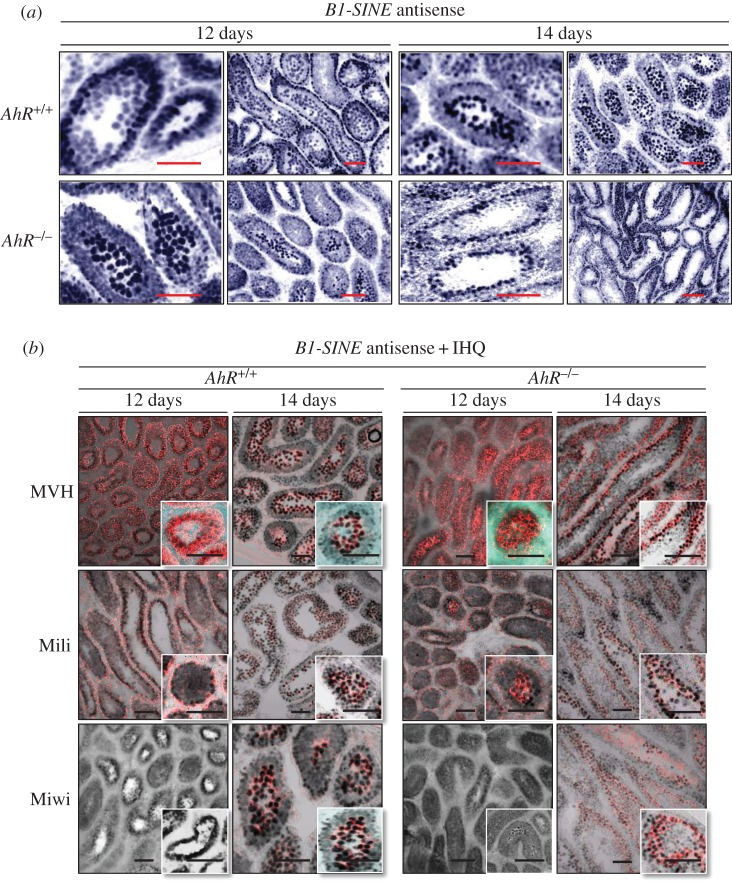
B1-SINE retrotransposons and piRNA-associated proteins have similar expression patterns in mouse testes. (*a*) *AhR^+/+^* and *AhR*^−/−^ testes obtained at 12 and 14 dpp were processed for *in situ* hybridization as indicated in Material and methods. Tissue sections were analysed for *B1-SINE* expression using the antisense sequence. (*b*) *In situ* hybridization for the *B1-SINE* retrotransposon was combined with immunofluorescence for MVH, Mili and Miwi in testis sections from *AhR^+/+^* and *AhR*^−/−^ mice at 12 and 14 dpp. Details of the micrographs are shown in the insets. *In situ* hybridization is shown in black in panel (*b*) to emphasize MVH, Miwi and Mili protein expression. Three biological replicates were used for each experiment. Scale bar, 50 µm.

Altogether, these results suggest that transposons and piRNA-associated proteins are co-expressed in an AhR-dependent manner during murine spermatogenesis. To address this possibility, we combined *in situ* hybridization for *B1-SINE* retrotransposons (as a representative family of repetitive elements in the testis) with immunofluorescence for MVH, Mili and Miwi proteins. A consensus sequence for the *B1-SINE* retrotransposon was cloned and used to synthesize sense (SE) and antisense (AS) probes (electronic supplementary material, figure S1*a*). The specificity of the hybridization reaction was confirmed by the negligible signal produced by the sense probe in 12 dpp male mice testis (electronic supplementary material, figure S1*b*). *In situ* hybridization showed that *AhR^+/+^* testes expressed *B1-SINE*-derived non-coding RNA transcripts at the outer spermatogonia layer in 12 dpp seminiferous tubules and at the inner spermatocyte compartment at 14 dpp (figure 5*a*, top). By contrast, seminiferous tubules from *AhR*^−/−^ mice already had *B1-SINE*-derived transcripts in the spermatocyte zone at 12 dpp that moved to the spermatogonia compartment at 14 dpp (figure 5*a*, bottom). Combined *in situ* hybridization and immunofluorescence revealed that MVH decorated the same cells expressing *B1-SINE* transcripts at either 12 or 14 dpp with a genotype-specific pattern (figure 5*b*, top). Mili had lower expression levels than MVH in *AhR^+/+^* and *AhR*^−/−^ testes, but its pattern of expression was coincident with that of *B1-SINE* transposons (figure 5*b*, middle). Miwi was only detected at 14 dpp, and its expression was coincident with *B1-SINE* positive spermatocytes in *AhR^+/+^* testis and with *B1-SINE* positive cells at the spermatogonia layer in *AhR*^−/−^ mice (figure 5*b*, bottom). These data suggested that AhR is involved in a mechanism regulating the levels of retrotransposons, piRNA-associated proteins and piRNAs during mouse spermatogenesis.

**Figure 6.**
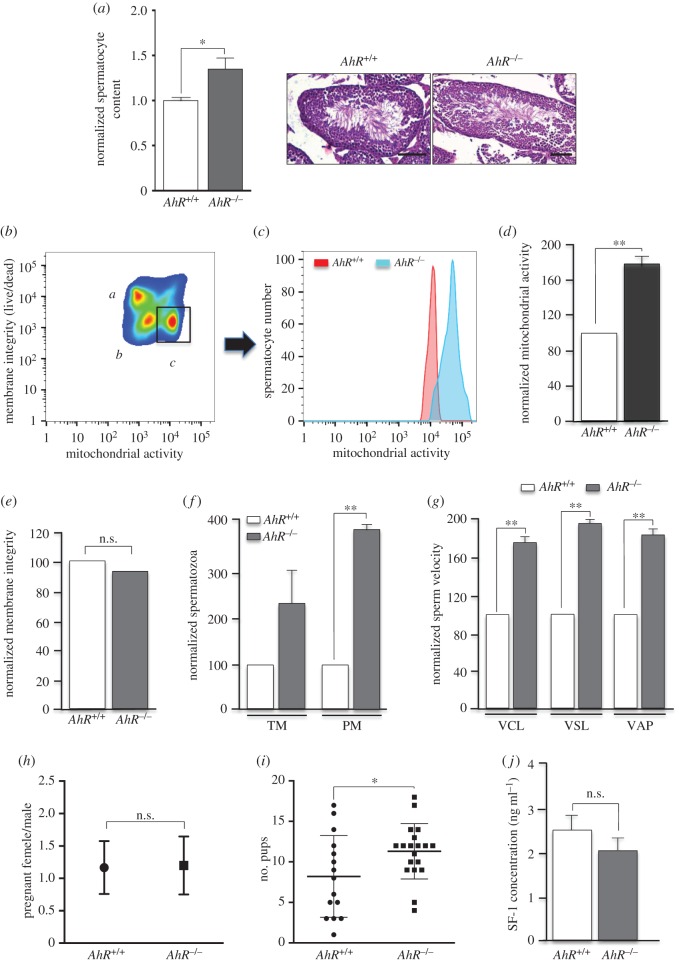
Young mice lacking AhR have increased sperm counts and enhanced fertility potential *in vivo*. (*a*) Epididymides were obtained from eight to 10-week-old male *AhR^+/+^* and *AhR*^−/−^ mice and the number of viable sperm cells measured in Pure Sperm Wash medium. Each sample was counted by two independent observers taking at least eight independent measurements. Representative sections of testis from *AhR^+/+^* and *AhR*^−/−^ mice stained with haematoxylin–eosin are shown. (*b*) Membrane integrity and mitochondrial membrane potential were assessed using flow cytometry. A representative cytogram depicting three sperm populations of dead spermatozoa (*a*), live spermatozoa with low mitochondrial membrane potential (*b*) and live spermatozoa with high membrane potential (*c*) is shown. (*c*) Hierarchical gating applied to the region of spermatozoa with high membrane potential and representative overlay histogram comparing mitochondrial membrane potential between *AhR^+/+^* and *AhR*^−/−^ mice. (*d*) Normalized mitochondrial membrane potential with respect to the values found in wild-type mice. (*e*) Measurement of membrane integrity in spermatozoa of both genotypes. (*f*) Percentages of total motile (TM) and progressive motile (PM) spermatozoa normalized with respect to the values found in *AhR^+/+^* mice. (*g*) Spermatozoa velocities, circular (VCL) straight line (VSL) and average (VAP), in *AhR^+/+^* and *AhR*^−/−^ mice. Measurements were obtained in μm s^−1^. Sperm functionality was analysed using the Computer-Assisted Sperm Analysis software (CASA). (*h,i*) *AhR^+/+^* and *AhR*^−/−^ male mice (at least four of each genotype) were breed with ICR females at six to eight weeks of age (20 per genotype). The number of pregnant females per male was determined (*h*). Viable pups were recovered at 18 dpc from pregnant females bred with either *AhR^+/+^* or *AhR*^−/−^ male mice (*i*). (*j*) The steroidogenic marker SF-1 was analysed in *AhR^+/+^* and *AhR*^−/−^ male mouse serum using an ELISA kit as indicated by the manufacturer. Data in panels (*a*) and (*d–i*) are shown as means ± s.d. *p < 0.05, **p < 0.01. n.s., not statistically significant. Adult mice were 12–13 weeks of age. Scale bar, 50 µm.

We then asked if these phenotypes produced by AhR deficiency have functional relevance considering that they were observed in young fertile male mice. First, epididymis was recovered from eight- to 10-week-old young mice and their content in viable sperm determined. *AhR*^−/−^ mice produced significantly more viable spermatocytes than wild-type littermate mice (figure 6*a*). We then decided to investigate differences in spermatozoa function between both genotypes. Flow cytometry analysis revealed that, within the population of live sperm with high mitochondrial membrane potential (figure 6*b*), the total mitochondrial activity was significantly higher in *AhR*^−/−^ spermatozoa than in littermate *AhR^+/+^* sperm (figure 6*c,d*). Yet, the integrity of the membrane in epididymal sperm was not affected by AhR expression (figure 6*e*). Notably, the numbers of TM and progressive motile (PM) sperm were markedly higher in *AhR*^−/−^ than in *AhR^+/+^* mice (figure 6*f*). Moreover, additional sperm motility parameters such as the circular (VCL), straight (VSL) and average (VAP) velocities revealed that AhR-null sperm moved significantly faster than wild-type spermatocytes from littermate mice (figure 6*g*). Based on these functional data, we decided to perform breeding experiments of *AhR^+/+^* and *AhR*^−/−^ male mice with fertile eight- to 10-week-old ICR female mice. We found no significant difference in the number of pregnant ICR females bred with either *AhR^+/+^* or *AhR*^−/−^ male mice (figure 6*h*). Interestingly, however, the number of viable pups recovered at 18 dpc from pregnant ICR females was significantly higher when bred with AhR-null males than when bred with wild-type AhR males (figure 6*i*). Because AhR has been associated with steroidogenesis in the testis [10], we have performed a preliminary analysis of steroidogenic activity in *AhR^+/+^* and *AhR*^−/−^ mice serum using SF-1 as surrogate marker. As shown in figure 6*j*, SF-1 levels were not significantly altered by AhR depletion, suggesting that steroidogenesis may not have a major role in the increased fertility of AhR-null male mice.

### 3.3. Female mice lacking aryl hydrocarbon receptor have reduced expression of transposons and low levels of piRNA-associated proteins

**Figure 7.**
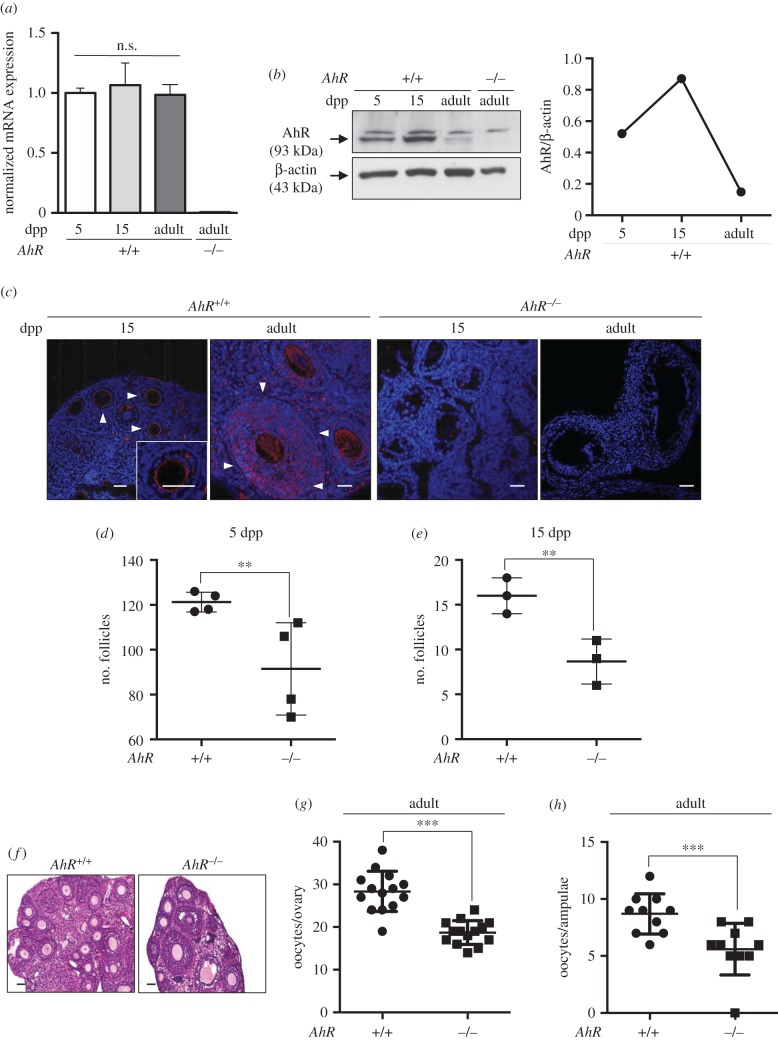
AhR deficiency reduces the number of follicles in the ovary. (*a*) Ovaries were extracted from 5 and 15 dpp, and adult (six to eight weeks) *AhR^+/+^* mice and used to obtain total RNA. *AhR* mRNA expression was quantified by RT-qPCR as indicated in Material and methods. Gene expression was normalized by *Gapdh* and represented as 2^−ΔΔCt^. Ovaries from adult *AhR*^−/−^ mice were used as negative control. (*b*) Ovaries from *AhR^+/+^* mice at the ages indicated above were used to prepare total protein extracts. AhR protein expression was determined by immunoblotting as indicated in Material and methods. Ovaries from adult *AhR*^−/−^ mice were used as negative control. (*c*) *AhR^+/+^* and *AhR*^−/−^ ovaries were processed for immunofluorescence and stained with an AhR-specific antibody. Alexa-633-labelled secondary antibody was used to detect AhR expression. DAPI staining was added to label cell nuclei. Arrowheads mark AhR expression. (*d*,*e*) The number of follicles was quantified in 5 (*d*) and 15 dpp (*e*) *AhR^+/+^* and *AhR*^−/−^ ovaries. (*f*) Haematoxylin–eosin staining was performed to analyse the follicles present in adult *AhR^+/+^* and *AhR*^−/−^ ovaries. (*g,h*) Oocytes were extracted from the ovary (*g*) or the ampulae of the oviduct (*h*) of adult mice of both genotypes. At least five biological replicates were analysed for each genotype. Duplicate or triplicate experimental determinations were done. Data in panels (*a,d,e,g,h*) are shown as means ± s.d. **p < 0.01, ***p < 0.001. n.s., not statistically significant. Adult mice were 12–13 weeks of age. Scale bar, 50 µm.

AhR is involved in the homeostasis of the female reproductive system and its deficiency affects mouse and rat fertility [3]. Based on the observations made in the testis, we next investigated if the absence of AhR also affects the expression of transposons and piRNA-associated proteins in the ovary. Levels of AhR mRNA did not significantly change in the ovary from neonates until the adult age (six to eight weeks; figure 7*a*). However, AhR protein expression moderately increased from the pre-antral stage at 5 dpp to the early antrum formation phase at 15 dpp, to then rapidly decrease in adult ovaries (figure 7*b*). Immunohistochemical analyses for AhR in 15 dpp wild-type mouse ovary showed receptor expression at the periphery of the oocyte in the early follicle zone. In adult *AhR^+/+^* ovaries, AhR was not only expressed in oocytes, but also in the surrounding layers of granulosa cells (figure 7*c*). Controls using 15 dpp and adult *AhR*^−/−^ mice showed no detectable AhR expression in the ovary (figure 7*c*). AhR deficiency caused a significant decrease in the number of pre-antral follicles (5 dpp), early antral follicles (15 dpp) and in adult female ovaries when compared with *AhR^+/+^* mice (figure 7*d*–*f*). Moreover, fewer numbers of oocytes were isolated from the ovary (figure 7*g*) and the ampulae (figure 7*h*) in adult AhR-null mice.

**Figure 8.**
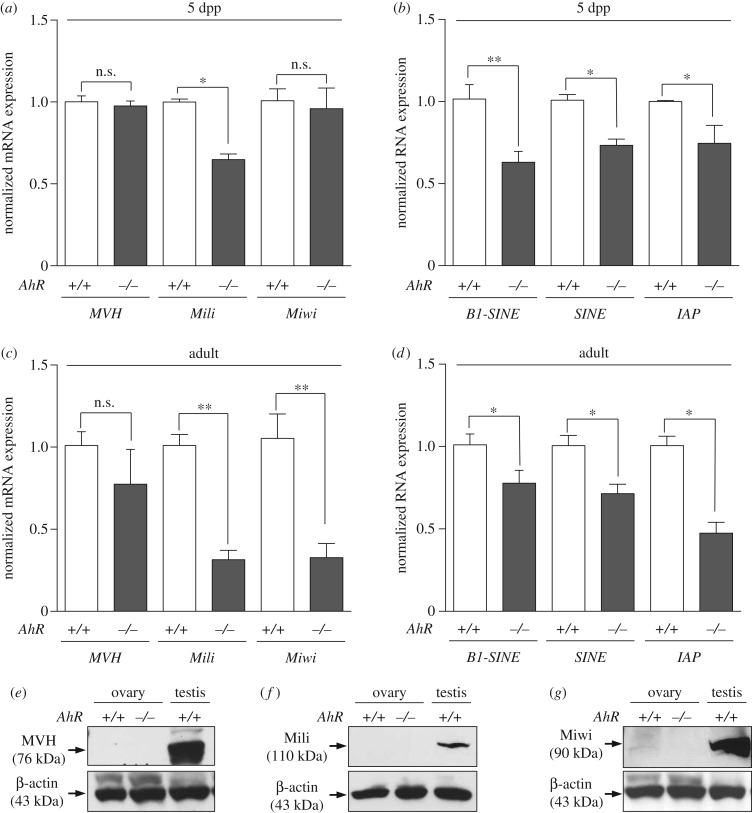
AhR deficiency reduces the levels of transposons and piRNA-associated proteins in the ovary. (*a*) Total RNA was isolated from ovaries of 5 dpp *AhR^+/+^* and *AhR*^−/−^ mice and used to analyse by RT-qPCR the mRNA expression of piRNA-associated proteins *MVH*, *Mili* and *Miwi*. (*b*) Total RNAs were also used to quantify by RT-qPCR the levels of retrotransposons of the *SINE* and *IAP* families and *B1-SINE subfamily*. (*c*) Ovaries from adult (five to six weeks) *AhR^+/+^* and *AhR*^−/−^ mice were used to isolate total RNA. The mRNA levels of *MVH, Mili* and *Miwi* were determined by RT-qPCR. (*d*) Total RNAs were also used to analyse by RT-qPCR the expression of *B1-SINE*, *SINE* and *IAP* retrotransposons in adult *AhR^+/+^* and *AhR*^−/−^ ovaries. Gene expression has been normalized by *Gapdh* and represented as 2^−ΔΔCt^. (*e–g*) Protein expression of MVH, Mili and Miwi was analysed by immunoblotting using total ovary protein extracts from adult *AhR^+/+^* and *AhR*^−/−^ mice. Experiments were done in duplicate in four biological replicates. The expression of β-actin was used to normalize protein loading. Data in panels (*a–d*) are shown as mean ± s.d. *p < .05, **p < 0.01. n.s*.*, not statistically significant. Adult mice were 12–13 weeks of age.

**Figure 9.**
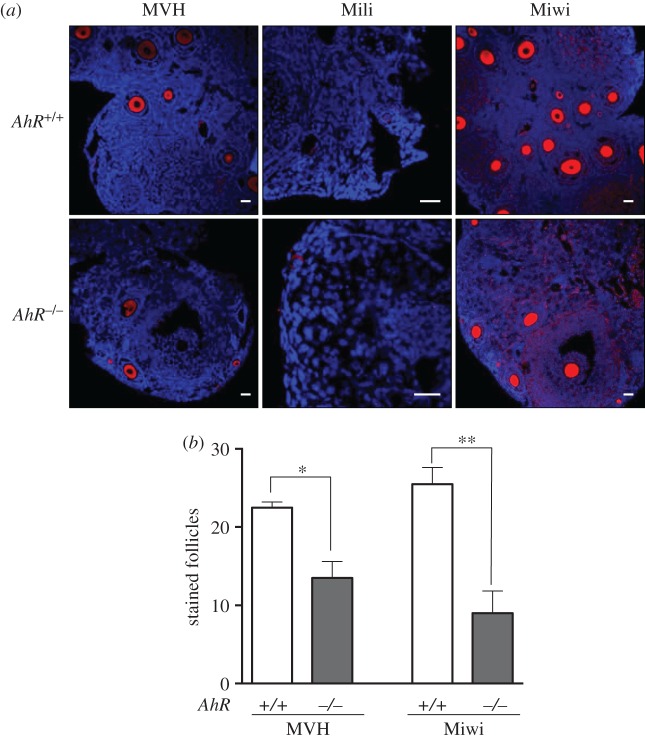
Adult AhR-null mice have reduced numbers of MVH- and Miwi-positive ovarian follicles. (*a*) Ovaries from adult (five to six weeks) *AhR^+/+^* and *AhR*^−/−^ mice were extracted and processed for the detection of MVH, Mili and Miwi by immunofluorescence. An Alexa-633-labelled secondary antibody has been used. (*b*) The number of positive follicles was quantified for each individual ovary of each mouse genotype. Ovaries from at least four *AhR^+/+^* and *AhR*^−/−^ mice were used, and immunofluorescences were done in triplicate. Data in panel (*b*) are shown as mean ± s.d. *p < 0.05, **p < 0.01. Adult mice were 12–13 weeks of age. Scale bar, 50 µm.

**Figure 10.**
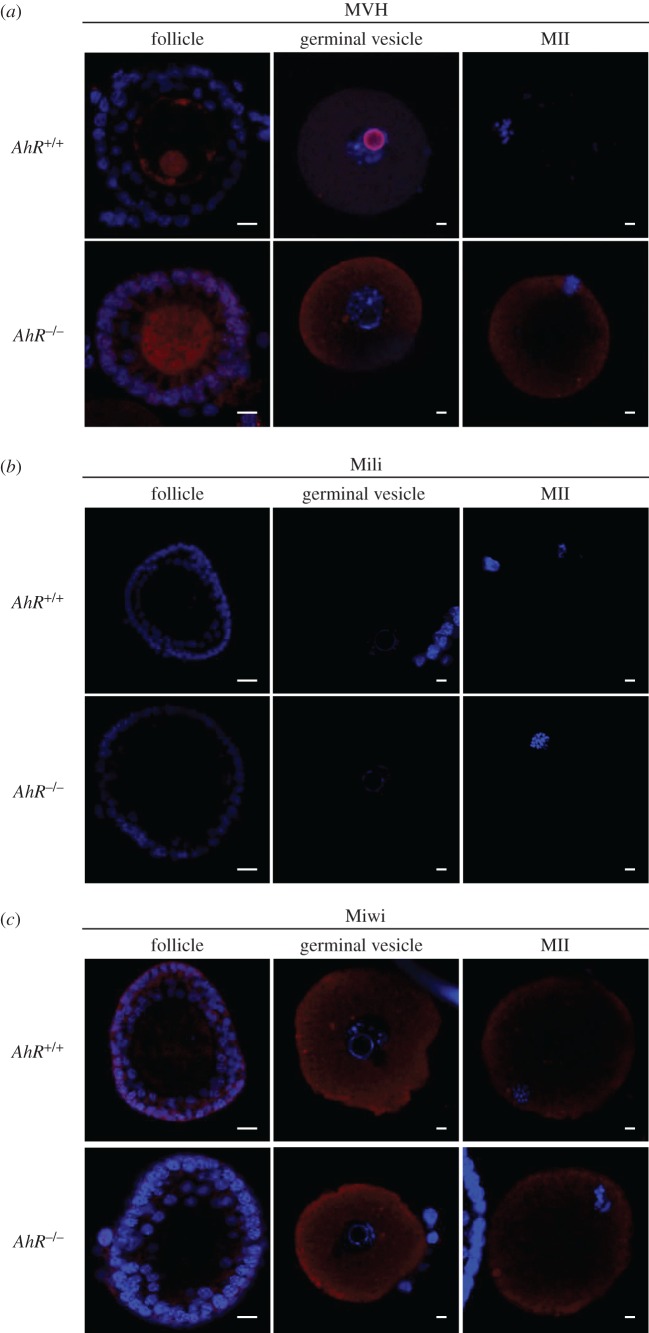
Lack of AhR alters the pattern of MVH expression during oocyte maturation. (*a–c*) Oocytes at the follicle, GV and MII stages were extracted from *AhR^+/+^* and *AhR*^−/−^ mice as indicated in Material and methods. The protein expression of MVH (*a*), Mili (*b*) and Miwi (*c*) was analysed by immunofluorescence using specific antibodies. An Alexa-633-labelled secondary antibody has been used. Groups of 10 mice of each genotype were used to extract oocytes at the different developmental stages. Adult mice were 12–13 weeks of age. Scale bar, 50 µm.

The female reproductive system is also exposed to the deleterious effects of retrotransposons. Nuage and piRNA-associated proteins are activated in the ovary early after birth (5 dpp) possibly to silence retrotransposons and retrotransposon-containing genes [29]. Ovaries isolated from 5 dpp *AhR*^−/−^ mice had significantly lower levels of *Mili* mRNA but showed no significant changes in the amounts of *MVH* and *Miwi* mRNAs (figure 8*a*). Retrotransposons of the *SINE* and *IAP* families and *B1-SINE* subfamily were also downregulated in 5 dpp *AhR*^−/−^ ovaries compared with those in *AhR^+/+^* mice (figure 8*b*). In adult AhR-null females (five to six weeks), a large decrease in *Mili* and *Miwi* mRNAs was observed (figure 8*c*) that concurred with a significant repression of *SINE, B1-SINE* and *IAP* transposons (figure 8*d*). *MVH* mRNA levels were not significantly altered by AhR deficiency in the adult ovary (figure 8*c*). Thus, AhR deficiency in the ovary results in an expression pattern of transposons and piRNA-associated proteins that appears opposed to that found in the testis. Immunoblot analysis of total ovary protein extracts revealed very low or undetectable levels of MVH, Mili and Miwi in adult *AhR^+/+^* and *AhR*^−/−^ mice (figure 8*e*–*g*). Nevertheless, immunofluorescence analysis showed that the number of follicles expressing MVH and Miwi in adult ovaries was significantly reduced in *AhR*^−/−^ mice when compared with *AhR^+/+^* mice (figure 9*a*,*b*), and that Mili expression was undetectable in ovaries of either genotype (figure 9*a*). Despite the reduced number of MVH- and Miwi-positive follicles present in *AhR*^−/−^ mice, the expression levels of both proteins in individual follicles were similar between genotypes, suggesting that a deficient formation of follicles could maintain lower total amounts of piRNA-associated proteins MVH and Miwi in the ovary. Based on these results, we next analysed the expression of MVH, Mili and Miwi at different stages of ovarian maturation corresponding to follicles, GVs and MII oocytes. MVH expression was maintained in *AhR*^−/−^ oocytes during the transition from follicle to GV to MII oocytes (figure 10*a*). By contrast, MVH was expressed at reduced levels in follicles and GVs of *AhR^+/+^* oocytes and it was undetectable in MII oocytes (figure 10*a*). Interestingly, MVH was expressed in granulosa cells of *AhR*^−/−^ but not of *AhR^+/+^* follicles (figure 10*a*). Mili and Miwi did not show significant differences in expression in follicles, GVs and MII oocytes between *AhR^+/+^* and *AhR*^−/−^ mice (figure 10*b*,*c*).

## 4. Discussion

The molecular complexes formed by Piwi proteins and piRNAs have a major role in transposon silencing during germline development with the aim to ensure genome integrity and stability in mature spermatocytes and oocytes [19,20,26,40,42]. Mili, Miwi and Miwi2 are needed for mouse spermatogenesis and, as a consequence, mice lacking Mili and Miwi2 are sterile and have increased retrotransposon expression in the germline [22–24]. Interestingly, AhR controls the transcription of murine *B1-SINE* retrotransposons whose upregulation represses differentiation-related genes [30,31,43].

AhR has pleiotropic effects in the male and female reproductive systems, but the signalling pathways and mechanisms involved remain largely unknown [3]. Whereas AhR depletion in female mice seems to affect their ovarian development and fertility [3,17], the role of AhR in the reproductive system of young male mice remains poorly understood. In this study, we have investigated the contribution of AhR to the maturation of germ cells in young male and female mice, focusing on the possible functional interaction between piRNA-associated proteins, piRNAs and retrotransposons. We conclude that AhR deficiency exerts an impact on the expression profile of piRNA-associated proteins, piRNAs and retrotransposons in the testis and ovary, and that such organ-specific profiles may be associated with or cooperate with a general developmental alteration in oogenesis and spermatogenesis that would finally affect fertility of female and male AhR-null mice. Taking into account the complex role of AhR in gonads development and maintenance [3,12], we cannot exclude that the mechanism proposed here could be integrated or result from a broader phenotype in which AhR deficiency affects not only tissue and cell homeostasis in the testis and ovary but also endocrine, metabolic, behavioural and sexual aspects of reproduction.

The implication of AhR in controlling the expression of piRNA and piRNA-associated proteins in testis was first supported by the pattern of receptor expression in young male mice. AhR protein levels peaked in testis from late leptonema (10 dpp) to pachynema (14 dpp), a developmental window during which most transposon activity has been detected and when pre-pachynema and pachynema piRNAs are produced to block adverse transposon effects [26,44,45]. AhR has a repressive role in the control of transposon expression during these critical stages of the male germline, because the levels of several well-conserved families of murine retrotransposons were significantly upregulated in *AhR*^−/−^ testes having pre-pachytene and pachytene spermatocytes, among them, members of the *B1-SINE* family of retroelements that are regulated by this receptor [30,31,43]. In agreement with our hypothesis, processed *B1-SINE* transcripts were more abundant in *AhR*^−/−^ than in littermate *AhR^+/+^* testes from 8 to 14 dpp, as were the amounts of processed piRNAs expressed during these early maturation stages. These results suggest that the endogenous expression of piRNAs and retrotransposons must be coordinated in wild-type testis. By contrast, in AhR-null testis, an increase in retrotransposon-derived transcripts takes place concomitantly with elevated levels of piRNAs, which could be interpreted as an enhanced protecting activity to ameliorate the negative effects of transposon-derived transcripts. Thus, AhR may have regulatory functions in controlling retrotransposons and piRNAs to allow proper spermatocyte development.

MVH is expressed in the mouse from leptonema (10.5 dpp) to the round spermatid stage (20 dpp) [25]. MVH cooperates with germline-specific Miwi, Mili and Miwi2 in transposon silencing through piRNA amplification in the early stages of spermatocyte and oocyte maturation [22–24,29]. The fact that MVH and Miwi, and to a lesser extent Mili, were overexpressed in *AhR*^−/−^ spermatocytes and adult testes supports the implication of AhR in the control of piRNA-associated proteins. Interestingly, MVH and Miwi had an unusual distribution in the seminiferous tubules in the absence of AhR. Whereas MVH and Miwi were expressed in the outer layer of spermatogonia-containing cells in leptonema and pre-pachynema *AhR^+/+^* testes, both proteins were mainly present in the inner compartment of differentiated spermatocytes in *AhR*^−/−^ tubules, suggesting that AhR depletion could anticipate, or to some extent accelerate, the process that contributes to the maturation of male germ cells. The pattern of piRNA-associated proteins in *AhR*^−/−^ testis was indeed coincident with higher levels of retrotransposon expression. Notably, MVH, Mili and Miwi were co-expressed and co-localized with transcripts from AhR-regulated *B1-SINE* retrotransposons in the spermatogonia-containing layer in pre-pachytene 12 dpp *AhR^+/+^* mice but with the spermatocyte compartment in *AhR*^−/−^ mice. This genotype-dependent co-localization of *B1-SINE* transcripts and piRNA-associated proteins suggests that AhR-null pre-pachytene spermatocytes are already exposed to transposon-derived transcripts at 12 dpp, whereas *AhR^+/+^* spermatocytes will not be exposed until the pachytene stage at 14 dpp. Whether such accelerated process has a causal role or a direct influence in sperm maturation in *AhR*^−/−^ mice remains to be investigated. Certainly, *AhR^+/+^* spermatocytes show the same co-distribution between piRNA-associated proteins and *B1-SINE* transcripts, although at a latter developmental stage. We suggest that AhR may be an intermediate molecule in the mechanism controlling physiological levels of transposon-derived transcripts, piRNA-associated proteins and piRNAs during the maturation of male germ cells.

Nuage proteins are essential for male mice fertility since loss-of-function mutations in their coding genes deregulate spermatogenesis [28]. Contrary to the lower sperm counts reported in old *AhR*^−/−^ mice aged up to 52 weeks [10], we found that young *AhR*^−/−^ male mice produced an increased number of sperm cells with higher mitochondrial activity and improved motility compared with those from littermate *AhR^+/+^* males. That early study [10] did not report, however, significant differences in testis weight, seminal vesicle regression or sperm content in younger 24-week-old AhR-null mice. The apparently healthier properties of AhR-null spermatocytes reported here are consistent with the higher fertility rate obtained when mating *AhR*^−/−^ male mice with standard ICR females. A previous study showed that C57BL/6-Ahr^m1.2Arte^ null-mice (Taconic) have an accumulation of elongated spermatids and reduced fertility compared with wild-type mice in *in vitro* fertilization assays [36]. This difference could be explained by the fact that the *in vivo* assay used in our study may be a more physiological approach to determine differences in mice fertility because it is known that the mating itself and the different molecules (hormones and growth factors among others) that altogether constitute the sperm have a relevant impact in mice fertility [46,47]. In addition, the former study used C57BL/6JAhr^b-1^ as wild-type controls (Jackson Labs), and their different genetic background with respect to C57BL/6-Ahr^m1.2Arte^ null-mice could have influenced the *in vitro* phenotypes found. Recent studies using different physiological and molecular strategies have recognized the existence of high-fertility male mice phenotypes that are probably attributable to the accumulation of endocrine (hormonal levels, e.g. testosterone), physiological (accelerated puberty) and behavioural advantages [46,47]. We therefore suggest that the fertility of *AhR*^−/−^ male mice may depend on improved sperm functionality and on endocrine, physiological and/or behavioural advantages due to AhR depletion. In this regard, an initial analysis of steroidogenesis revealed that the steroidogenic activity present in AhR-null mice serum was very similar to that of AhR wild-type mice, suggesting that cholesterol-derived steroid hormones may not have a prominent role in the enhanced fertility of *AhR*^−/−^ male mice. In agreement, a former study indicated that whereas the level of the steroidogenic regulator StAR (steroidogenic acute regulatory gene) was reduced, those of SF-1, P450scc (side chain cleavage) and insulin like-3 (Insl3) remained unchanged in 24- and 52-week-old *AhR*^−/−^ male mice [10], indicating that this pathway could not have a determinant role in the observed phenotype. Additionally, an earlier expression pattern of piRNA and piRNA-associated proteins may also give an advantage to *AhR*^−/−^ mice to produce more competent sperm cells.

The effects of AhR on the expression of retrotransposons and piRNA-associated proteins in the mouse ovary appeared opposite to those found in the testis. AhR deficiency severely impaired follicle development and reduced the number of oocytes in the ovary and ampulae. These observations concur with previous studies reporting reduced ovulatory potential and decreased fertility in *AhR*^−/−^ female mice [12,17,18]. Nuage proteins, such as MVH, and piRNA-associated proteins, such as Mili and Miwi, are also relevant for retrotransposon silencing in the ovary, although their de-repression does not seem to compromise fertility [29,48]. *AhR*^−/−^ ovaries had reduced expression of MVH and Miwi and lower levels of retrotransposon-derived transcripts in both postnatal and adult mice. The reduction in total levels of MVH and Miwi in adult *AhR*^−/−^ ovaries is more probably due to fewer numbers of follicles expressing normal amounts of these proteins than to a decrease in their expression in individual follicles. One possibility is that, considering the *AhR*^−/−^ ovary as a whole, fewer follicles expressing piRNA-associated proteins could compromise the organ's potential to silence retrotransposons. Whether such global reduction in MVH and Miwi has an active or causal role in follicle maturation remains unknown and should deserve further analysis. Interestingly, MVH deficiency can induce de-repression of transposons in primordial follicles, although, as indicated above, it does not seem to significantly affect female fertility [29]. The role of piRNA-associated proteins in oocyte development appears complex. *In vitro* maturation assays have revealed that MVH was present in *AhR^+/+^* oocytes at the follicle and GV stages, but not in MII oocytes. However, in *AhR*^−/−^ mice, MVH expression strongly persisted from follicles to MII oocytes, and it was also present in the granulosa cells of the follicle. The persistent MVH expression in *AhR*^−/−^ oocytes may be suggestive of a prolonged exposure to the deleterious effects of transposons, which could eventually affect their maturation. Unfortunately, transposon levels (e.g. *B1-SINE*) could not be determined by *in situ* hybridization during the *in vitro* maturation of follicles. Overall, we propose that AhR deficiency compromises female mice fertility by impairing oogenesis and reducing the number of viable follicles together with the possible deregulation of piRNA-associated proteins and retrotransposons.

In summary, we report here that AhR expression has an influence in piRNAs and piRNA-associated proteins in the testis and ovary of young mice in an organ-specific manner, and that such a process seems to correlate with altered retrotransposons expression. AhR deficiency results in increased expression of retrotransposons, piRNAs and piRNA-associated proteins during male germ cell maturation. Such a deregulated process could accelerate the maturation of spermatocytes and eventually increase male fertility. In the ovary, lack of AhR produces an opposite phenotype with reduced expression of piRNA-associated proteins and retrotransposons during early and late development. These alterations, together with an inefficient/ unproductive oogenesis, could eventually reduce the number of viable follicles and compromise female mice fertility. Although the mechanisms by which AhR intervenes in the crosstalk between retrotransposons, piRNAs and piRNA-associated proteins in germ cells deserve further investigation, we suggest that such signalling may have a relevant role in the maturation of spermatocytes and oocytes.

## Supplementary Material

Supplementary Figure 1

## Supplementary Material

Supplementary Table 1

## Data accessibility

Raw data used in this study to determine gene and transposon expression levels, sperm functionality and steroidogenesis are available at https://figshare.com/s/5289689749757e0b1dda.
