# Supplementary material for: piRNA-associated proteins and retrotransposons are differentially expressed in murine testis and ovary of aryl hydrocarbon receptor deficient mice
Source: Open Biol. 2016 Dec 21;6(12):160186. doi: 10.1098/rsob.160186 (PMC5204120; doi:10.1098/rsob.160186)
Supplement: Supplementary Figure 1 [file rsob160186supp1.pptx]

## Slide 1
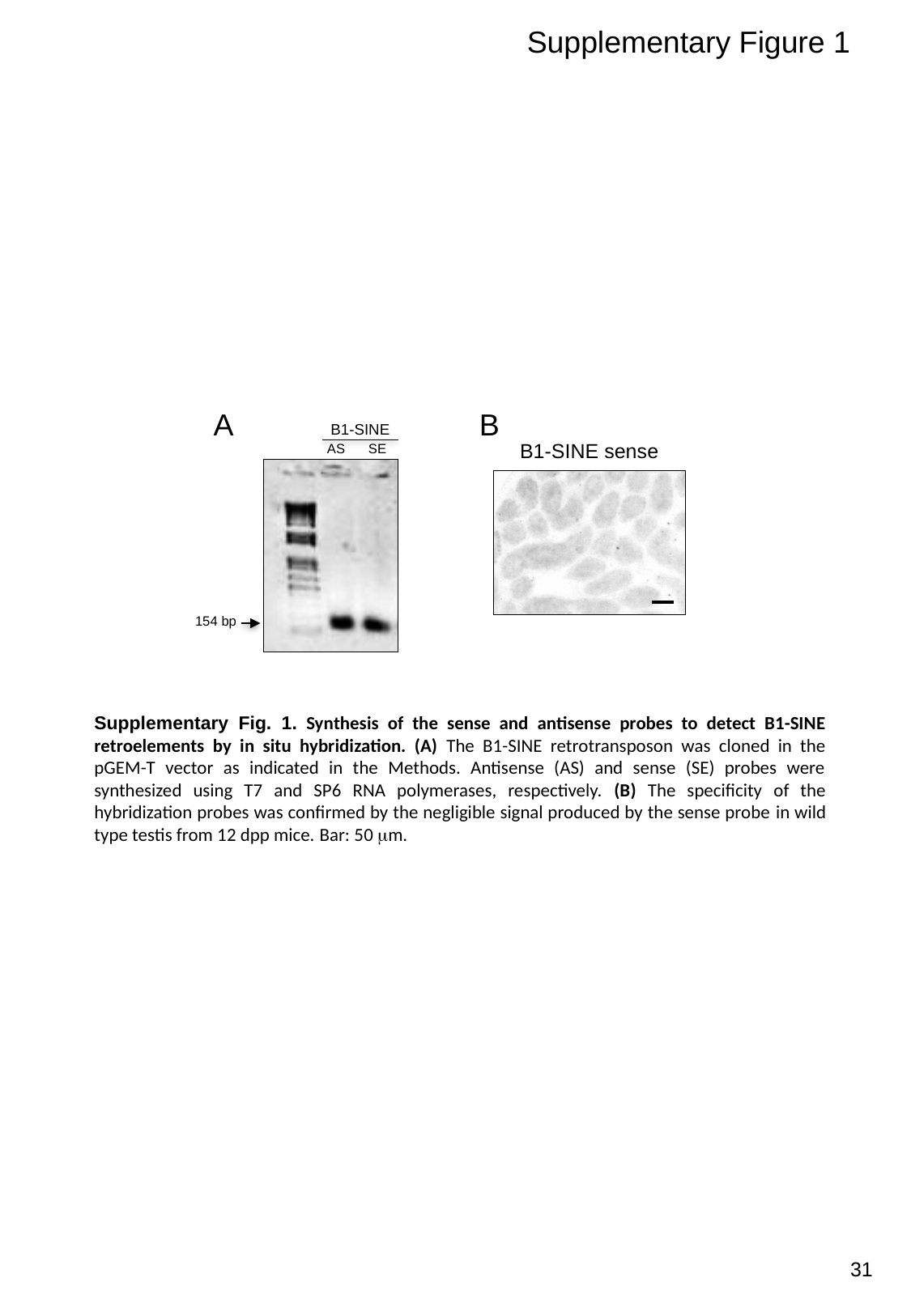

Supplementary Figure 1
A
B
B1-SINE
AS SE
154 bp
B1-SINE sense
Supplementary Fig. 1. Synthesis of the sense and antisense probes to detect B1-SINE retroelements by in situ hybridization. (A) The B1-SINE retrotransposon was cloned in the pGEM-T vector as indicated in the Methods. Antisense (AS) and sense (SE) probes were synthesized using T7 and SP6 RNA polymerases, respectively. (B) The specificity of the hybridization probes was confirmed by the negligible signal produced by the sense probe in wild type testis from 12 dpp mice. Bar: 50 mm.
31
