# Supplementary material for: piRNA-associated proteins and retrotransposons are differentially expressed in murine testis and ovary of aryl hydrocarbon receptor deficient mice
Source: Open Biol. 2016 Dec 21;6(12):160186. doi: 10.1098/rsob.160186 (PMC5204120; doi:10.1098/rsob.160186)
Supplement: Supplementary Table 1 [file rsob160186supp2.docx]

***Supplementary Table 1***

*Primer sequences for qPCR mRNA expression*

Gene name Primer sequence (5´- 3´)

*AhR* Forward: AGCCGGTGCAGAAAACAGTAA

Reverse*:* AGGCGGTCTAACTCTGTGTTC

*Mvh* Forward: ACCAAGATCAGGGGACACAG

Reverse: TAACCACCTCGACCACTTCC

*Mili* Forward: ACAAATGGTCGTTTGCATCA

Reverse*:* TTCAGCGGAATATCCACTCC

*Miwi* Forward*:* ATGGTAGTCGGAGCCACATC

Reverse*:* GGTGCTCAGCTTCACAATGA

*Cyp1a1* Forward*:* ACAGACAGCCTCATTGAGCA

Reverse: GGCTCCACGAGATAGCAGTT

*Gapdh* Forward*:* TGAAGCAGGCATCTGAGGG

Reverse*:* CGAAGGTGGAAGAGTGGGAG

*β-Actin* Forward*:* TGTTACCAACTGGGACGACA

Reverse*:* GGGGTGTTGAAGGTCTCAAA

*Primer sequences for transposons expression*

Gene name Primer sequence (5´- 3´)

*SINE* Forward: ACGCCTTTAATCCCAGCAC

Reverse*:* CTGGCCTCGAACTCAGAAAT

*B1-SINE* Forward*:* GTGGCGCACGCCTTTAATC

Reverse*:* GACAGGGTTTCTCTGTGTAG

*IAP* Forward*:* TTGGCAGATAAGGCCACTAAA

Reverse: ATTTCTTGCAGCCTCTACCG

*LINE1 5´UTR* Forward*:* GGCGAAAGGCAAACGTAAGA

Reverse: GGAGTGCTGCGTTCTGATGA

*LINE1 ORF2* Forward*:* GGAGGGACATTTCATTCTATCA

Reverse: GCTGCTCTTGTATTTGGAGCATAGA

*Primer sequences to amplify the B1-SINE for in situ hybridization*

Gene name Primer sequence (5´- 3´)

*B1-SINE* Forward*:* CTCGAGCTGCAGGCCGGGCGTGGTGGC

Reverse*:* CTCGAGCTGCAGCGAGACAGGGTTTCT
